# A new high-speed visual stimulation method for gaze-contingent eye movement and brain activity studies

**DOI:** 10.3389/fnsys.2013.00024

**Published:** 2013-07-01

**Authors:** Fabio Richlan, Benjamin Gagl, Sarah Schuster, Stefan Hawelka, Josef Humenberger, Florian Hutzler

**Affiliations:** ^1^Centre for Neurocognitive Research and Department of Psychology, University of SalzburgSalzburg, Austria; ^2^HTBLA LeondingLinz, Austria

**Keywords:** eye fixation-related potential, eye tracking, EEG, ERP, fMRI, projector, gaze-contingent display changes

## Abstract

Approaches using eye movements as markers of ongoing brain activity to investigate perceptual and cognitive processes were able to implement highly sophisticated paradigms driven by eye movement recordings. Crucially, these paradigms involve display changes that have to occur during the time of saccadic blindness, when the subject is unaware of the change. Therefore, a combination of high-speed eye tracking and high-speed visual stimulation is required in these paradigms. For combined eye movement and brain activity studies (e.g., fMRI, EEG, MEG), fast and exact timing of display changes is especially important, because of the high susceptibility of the brain to visual stimulation. Eye tracking systems already achieve sampling rates up to 2000 Hz, but recent LCD technologies for computer screens reduced the temporal resolution to mostly 60 Hz, which is too slow for gaze-contingent display changes. We developed a high-speed video projection system, which is capable of reliably delivering display changes within the time frame of < 5 ms. This could not be achieved even with the fastest cathode ray tube (CRT) monitors available (< 16 ms). The present video projection system facilitates the realization of cutting-edge eye movement research requiring reliable high-speed visual stimulation (e.g., gaze-contingent display changes, short-time presentation, masked priming). Moreover, this system can be used for fast visual presentation in order to assess brain activity using various methods, such as electroencephalography (EEG) and functional magnetic resonance imaging (fMRI). The latter technique was previously excluded from high-speed visual stimulation, because it is not possible to operate conventional CRT monitors in the strong magnetic field of an MRI scanner. Therefore, the present video projection system offers new possibilities for studying eye movement-related brain activity using a combination of eye tracking and fMRI.

## Introduction

The combined recording and analysis of eye movements and brain activity is one of the most promising developments in neuroscience (see Eye Movement-Related Brain Activity During Perceptual and Cognitive Processing). Recent studies used eye movements as markers for brain responses related to perceptual and cognitive processes during reading (e.g., Baccino and Manunta, 2005; Hutzler et al., 2007; Simola et al., 2009; Dimigen et al., 2011, 2012; Richlan et al., 2013), visual search (e.g., Healy and Smeaton, 2011; Kamienkowski et al., 2012), object identification (e.g., Rämä and Baccino, 2010; Marsman et al., 2012), and scene perception (e.g., Graupner et al., 2011; Nikolaev et al., 2011). Eye-movement-based research has particularly benefitted from the implementation of gaze-contingent display change paradigms such as moving window (McConkie and Rayner, 1975), moving mask (Rayner and Bertera, 1979), and invisible boundary paradigm (Rayner, 1975). The core of these paradigms is a display change during the very short time of a saccade. In the present paper, we present a novel high-speed visual stimulation system facilitating gaze-contingent display change paradigms, which replaces currently used but no longer produced cathode ray tube (CRT) monitors. Besides execution of extremely fast display changes for various kinds of visual experiments (e.g., involving gaze-contingent display changes, short-time presentation, masked priming), the main advantage of a projector-based system is its applicability in functional magnetic resonance (fMRI) experiments. This opens up a novel line of research of combined eye tracking and fMRI studies with the above-mentioned experimental paradigms. Our system enables the implementation of visual experiments in the fMRI scanner with the same temporal precision as outside the fMRI environment. This was previously not possible because of the incompatibility of CRT monitors with fMRI and the poor temporal properties of current MR-compatible LCD monitors and projectors.

Gaze-contingent paradigms rely on fast and exactly timed display changes in response to the participant's eye movement behavior. Crucially, the display changes have to occur during a saccade, when visual processing is suppressed and the participant is unaware of the change. The duration of the time window for this process depends on the amplitude of a saccade. To illustrate the time constraints on gaze-contingent display changes in natural reading (i.e., sentence or text reading), the amplitude of a typical saccade is about 6–7 letters (i.e., about 2 visual degrees), resulting in saccade durations of around 20–35 ms (Rayner, 2009; Slattery et al., 2011). Note that in most cases the invisible boundary is roughly in the middle between the starting position and the landing position of the saccade. Consequently, more than half of the saccade duration has already elapsed before the invisible boundary is crossed and this boundary crossing is detected by the eye tracker. Therefore, the display change should be completed within the short period of time of the second half of the saccade, immediately before the next fixations begins. As a consequence, fast timing and low variability is needed in order to guarantee that the majority of display changes take place during a saccade rather than during a subsequent fixation.

Using conditions that resemble existing studies, Slattery et al. (2011) showed that there can be irredeemable artifacts in the eye movement data when some of the display changes are not finalized before a subsequent fixation begins. Specifically, there is an interaction between timing of the display change and the amount and quality of information that is changed between the pre- and post-boundary stimulus. Critically, they found that the slower the display change was, the larger were the differences in the eye movement patterns. Thus, the effects of interest may be affected by slow display changes and may lead to a complex distortion of the experimental effects, which renders interpretation of findings difficult, if not impossible. Hence, for experiments employing gaze-contingent paradigms, it is crucial to deliver fast display changes during the time of saccadic blindness, when visual processing is suppressed. This can only be accomplished via rigorous control over the timing of display changes via a combination of high-speed eye tracking and high-speed visual stimulation. Especially for combined eye movement and brain activity studies, fast and exact timing of display changes is important, because of the high susceptibility of the brain to visual stimulation. Even if a delayed display change is not consciously perceived by the participant, it is likely to affect visual information extraction, which, in turn, may influence measures of brain activity (e.g., event-related potentials or hemodynamic responses).

In order to avoid display change artifacts in gaze-contingent paradigms, we developed a high-speed video projection system based on light-emitting diode (LED) technology. An LED-based tachistoscope was recently shown to provide a powerful means for extremely fast on-and-off switching of a visual display (Thurgood and Whitfield, 2013). This technology was proven useful in enabling minimal stimulus exposure durations for psychological experiments. Here, we extend this approach by presenting a system that should be capable of reliably delivering changes between two different visual displays in a similarly short time. The system is based on two converging projectors, which are toggled exactly at the moment when a boundary crossing is detected. This means that, rather than switching from one display to the next display with a single stimulation device (i.e., a monitor or a projector), we use two stimulation devices (i.e., two projectors), which are switched on and off, respectively, to change the display. Therefore, the display change can be realized independently from the projectors' LCD panel refresh rates (which are limited to 60 Hz). The present paper introduces and describes this high-speed video projection system. Furthermore, we present a hardware-based method for measuring the delay between the time point of the intended display change (i.e., immediately after the boundary crossing is detected by the eye tracker) and the actual display change. This method is based on a combination of a real-time photosensitive diode and an electroencephalography (EEG) amplifier. It can be used to assess the temporal properties of any visual stimulation setup (monitor or projector). For the present paper, we used this measurement circuit in order to assess the temporal properties of our newly developed video projection system in relation to a conventional CRT monitor with two refresh rates of 150 and 200 Hz. To do so, we implemented a typical gaze-contingent invisible boundary paradigm in a sentence reading task. We expected markedly faster display changes in our LED-based projector system compared to the CRT monitor for two reasons. First, the display change was controlled by a combination of the fast parallel port (Stewart, 2006) and a fast electronic circuit. Second, the projector setup was realized to bypass the process of building up a new display, which—in this context—is rather time consuming.

The present visual stimulation system should facilitate the realization of cutting-edge eye movement research requiring reliable high-speed visual stimulation in combined eye tracking and brain electrophysiological studies. It is not only applicable to experiments employing gaze-contingent display change paradigms but should also be feasible for short-time presentation and masked priming studies. In this context it replaces conventional CRT monitors. In addition, our system presents the necessary hardware features for a novel line of combined eye tracking and fMRI studies by enabling extremely fast visual presentation and implementation of gaze-contingent display change paradigms in the fMRI scanner. Fast visual presentation in the fMRI environment was previously not possible because of the poor temporal properties of LCD-based MR-compatible monitors and projectors.

## Materials and methods

### Participant

One participant conducted the sentence reading task in the projector setup measurement and both monitor setup measurements.

### Materials and procedure

**Figure 1:**
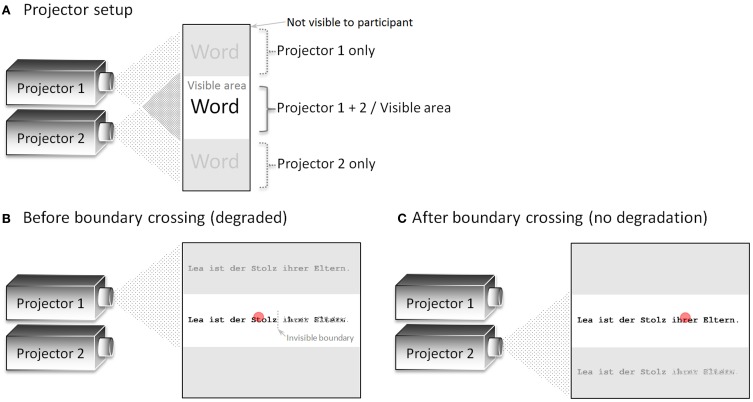
**(A)** Schematic depiction of the projector setup in general. Here, both projectors (placed behind a semi-transparent screen) are switched on to visualize the projection areas. **(B)** Presentation before the invisible boundary was crossed. Only the lower part of the display (containing the degraded sentence on the right-hand side of the invisible boundary) was visible to the participant. **(C)** Presentation after the invisible boundary was crossed. Only the upper part of the display (containing the un-degraded sentence) was visible to the participant. The red circle indicates the position of the eye of the participant.

A horizontal 3-point calibration routine preceded the experiment. Fixating between two vertical lines on the left margin of the screen triggered sentence presentation in such a way that the participant's fixation was at the center of the sentence's first word. One-hundred sentences from a currently conducted study were presented in black letters on a white background by the Experiment Builder software (SR-Research) in mono-spaced font (Courier New; single character width: ~0.3° of visual angle; see Figure 1 for an example). In each trial, a display change was initiated by a saccade from a pre-target to a target word, which was realized by the classical boundary paradigm (Rayner, 1975). To ensure fast display changes, we included a prepare sequence before each of our pre-built trials. Before the boundary crossing, all or some letters of the target word and all letters of the following words were degraded (i.e., about 45% of black pixels were displaced). When the eye crossed the invisible boundary, the display changed from the presentation of the degraded stimuli to a presentation without degradation. Sentence presentation was terminated after fixating an “X” in the lower right corner of the screen and recalibration was initiated in case the fixation control at the start of a trial failed. The target words were composed of five letters, were placed right after the invisible boundary, and were not predictable from sentence context. The invisible boundary and corresponding target words were never at the first, second, or last position of a sentence. In addition to the standard sentence presentation, which was vertically centered starting on the left of the screen, a black square (about 30° × 41°) was presented above the vertical center on the right end of the screen. This square triggered the measurement by the photosensitive diode. To specify, when the black square was presented, the photosensitive diode was switched off by the low amount of light that fell on the diode. After the invisible boundary was crossed, a white screen instead of the black square increased the light intensity presented to the photosensitive diode. The increase in light intensity reduced the resistance of the photosensitive diode, which consequently was switched on.

### Apparatus and setup

For all measurements, an EyeLink CL eye tracker (SR-Research, Canada) was used to record the movements of the right eye (at 2000 Hz). A forehead and chin rest stabilized the participant's head 52 cm in front of the monitor. For presentation of the stimuli, we used a PC with a Pentium 4 processor (2.8 GH processor speed), 2 GB RAM, a Nvidia GeForce 6200 graphics card, and a Windows XP operating system. In the following, two presentation setups were compared: the novel projector setup, which was realized with two projectors, and a monitor setup, which was realized with one of the fastest CRT monitor available.

#### Monitor setup

First, we used the state-of-the-art setup to estimate the latencies of gaze-contingent display changes for refresh rates of 150 and 200 Hz with the Vision Master Pro 454 monitor (Iiyama, Japan). For the 150 Hz refresh rate the display resolution was 1024 × 768 pixels, and for the 200 Hz refresh rate the display resolution was 640 × 480 pixels.

#### Projector setup

The projector setup is a new approach to present gaze-contingent displays. Two projectors (with a resolution of 1024 × 768 pixels), which were mounted on top of each other, behind a semi-transparent screen, were either switched on or off by an electronic circuit. The switching circuit allows very fast display changes despite the low refresh rate of the projectors' LCD units (60 Hz; display change latency about 45 ms as measured by the electronic circuit described in section Display Change Latency Measurement.). In the present experiment, we used an invisible boundary paradigm with one display change, which was realized by switching from one to the other projector. In order to be independent from the refresh rate of the projectors, the same display was presented by both projectors, with the lower part of projector 1 and the upper part of projector 2 converging at the center of a semi-transparent screen (Figure 1A). In the present paradigm, several words on the right-hand side of the invisible boundary were degraded prior to the display change. Therefore, projector 1 presented the degraded sentence in the lower part of the display, which was visible to the participant, before the boundary was crossed (Figure 1B). After the boundary was crossed, projector 1 was switched off and the un-degraded sentence was presented at the very same position by projector 2 (see Figure 1C). Importantly, only the area where the two projectors converged was visible to the participant sitting in front of the screen. In sum, the display change was realized by switching projector 1 off and projector 2 on at the moment the boundary was crossed. This on-and-off switching is independent of the actual refresh rate of the projectors and, therefore, results in faster display change latencies.

**Figure 2:**
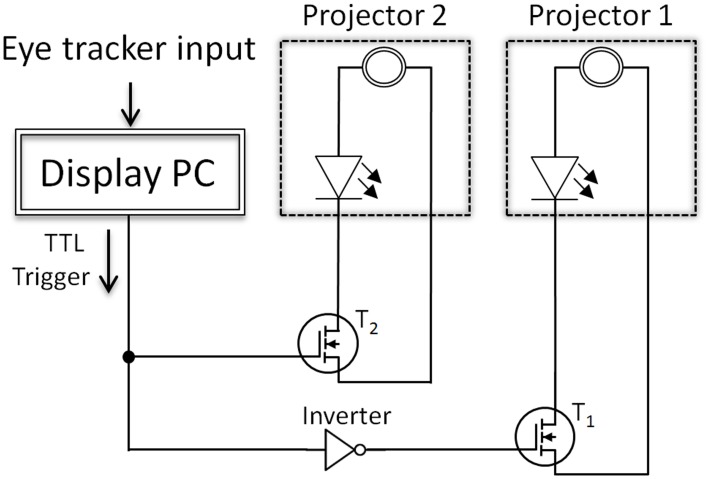
**Schematic depiction of the switching circuit**. *T*_1_ and *T*_2_ mark the power transistors that were used to switch the projectors either on or off. After the eye tracker indicated that the eye crossed the invisible boundary, the display change was initiated by a TTL trigger. This trigger was read out by the electronic circuit, which either connected or disconnected the projectors' LEDs from their original power sources. Note that this illustration is simplified and the actual circuit included six transistors—one for each LED of the two projectors.

In detail, the display change was controlled by the display PC (Figure 2). After the eye tracker indicated that the eye crossed the invisible boundary, the display change was initiated by a TTL trigger (latency between boundary cross and TTL trigger: between 1 and 2 ms). This trigger was read out by the electronic circuit and resulted in an immediate switching of the two projectors. The fast switching was possible as both projectors (SP-F10M, Samsung Electronics Co., Ltd., South Korea) used lighting based on LEDs, which were either switched on or off by transistors. As a consequence, the transistors either connected or disconnected the projectors' LEDs from their original power sources.

**Figure 3:**
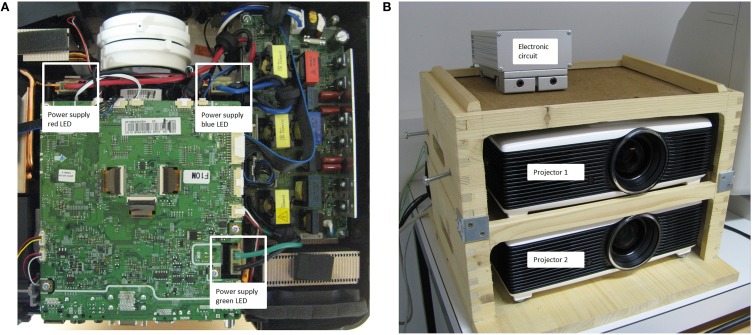
**(A)** The power plugs for the original power supplies of the red, blue, and green LEDs of the projector used in the present setup (Samsung SP-F10M). **(B)** The projectors were mounted in a wooden box with the electronic circuit (mounted in an aluminum box) on top.

Technically, the display changes of the projector setup were realized by a small but effective manipulation of the two projectors and a, rather simple, switching circuit. Figure 3A shows the top view of one projector with marked power supply ports of the three LEDs (red, blue, and green). Here, the original power cable connections of both identically constructed projectors were removed from their plugs. In order to control the power supply of the LEDs of the projector, the circuit presented in Figure 2 was applied in between the original power supply ports and cable connectors of the projectors. The dashed boxes in Figure 2 indicate the two projectors including a power supply and a LED. The cornerstones of the circuit were the power MOSFET transistors (BUZ 22) that were interposed between each LED of each projector and their original power sources. Note that for both projectors three transistors were used: one for each LED (red, blue, and green) of the projectors. For simplicity, Figure 2 presents only one power transistor for each projector, but the circuit was identical for all three LEDs. At the moment the eye crossed the invisible boundary, the parallel port of the display PC controlled the power transistors via TTL triggers. This TTL trigger raised a potential from 0 to 5 V at one data pin of the parallel port, which triggered a toggle between the projectors. This toggle was realized by an inverter (see Figure 2), which allowed, before the boundary was crossed and no TTL signal was present, that projector 1 was switched on (transistor *T*_1_ connected the power source and the LEDs) and projector 2 was switched off (transistor *T*_2_ disconnected the power source of the LEDs of projector 2). After the boundary crossing, the TTL trigger set the data pin to 5 V with the result that the power transistor of projector 1 was switched off by the inverter (i.e., signal inverted to 0 V) and the power transistor of projector 2, which was directly controlled by the parallel port, connected the power source of projector 2 to their LEDs. This on-and-off switching of the two projectors allowed extremely fast display changes despite the low refresh rate of the projectors (60 Hz). Figure 3B shows the two projectors mounted in a wooden box with the electronic circuit (mounted in an aluminum box) on top.

### Display change latency measurement

**Figure 4:**
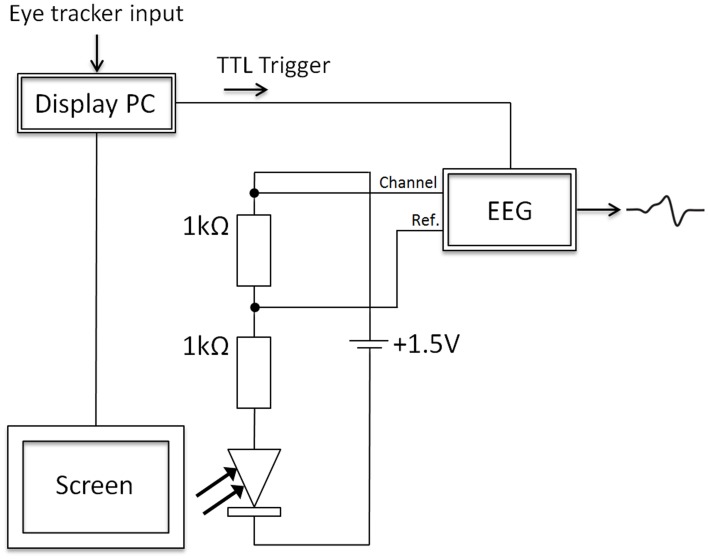
**The electronic circuit, which was used to measure the display change latency, involved two resistors (1 kΩ), a voltage source (battery; 1.5 V), and one photosensitive diode**. The diode was placed either on a black square on the monitor or in front of projector 2, which was switched off before the boundary was crossed. After the boundary cross, the light intensity at the photosensitive diode was increased by removing the black square or switching on projector 2. This difference in illumination was measured by the photosensitive diode and allowed assessing the display change latencies in all setups (projector, monitor 150 Hz, and monitor 200 Hz setups).

Another circuit was used to measure the display change latency after the eye crossed the invisible boundary (see Figure 4; for a similar measurement see Dorr, 2004). The cornerstone of this circuit was a photosensitive diode, which was placed either on the black square of the monitor setup or on projector 2. The monitor or projector 2 was used as light sources that were switched on at the boundary cross. After the boundary cross, the light intensity at the photosensitive diode was increased by removing the black square or switching on projector 2. This difference in illumination was measured by the photosensitive diode and allowed assessing the display change latencies of all the setups (projector, monitor 150 Hz, and monitor 200 Hz setups).

In Figure 4, the circuit is presented in detail. The display change latencies were measured by the combination of the electronic circuit (consisting of two resistors, a battery, and the photosensitive diode) and an EEG amplifier (BrainAmp MR+; sampling rate of 1000 Hz). Importantly, the photosensitive diode (SFH 203) decreased its resistance when the illumination at the diode increased. As a consequence, the voltage levels, which were measured by the EEG amplifier, increased. In the present experiment, before the invisible boundary was crossed, the diode was not illuminated and, therefore, had a high resistance (black square or projector LEDs were switched off). After the boundary was crossed, the black square was removed in the monitor setup or projector 2 was switched on. At this moment, the amount of light at the photosensitive diode increased, which resulted in a decrease of the resistance of the diode, and as a consequence voltage levels increased. In addition, at the time the boundary was crossed, a TTL trigger was sent to the EEG amplifier, which allowed referencing the signal from the photosensitive diode to the point in time when the boundary was crossed (as initiated by the display PC). Note that the TTL trigger was sent on a different data pin than the one used to toggle the projectors. The voltage measured by the EEG amplifier and the TTL trigger reference allowed estimating the display change latencies.

## Results

For the voltage change at the photosensitive diode circuit, the baseline correction was based on the 100 ms prior to the gaze-contingent display change (indicated by the TTL trigger). Furthermore, no signal processing filters were used, and due to absolute differences in voltage levels between the setups (projector setup: maximum of about 7 mV; monitor setup: maximum of about 2 mV), the voltage values were *z*-transformed. The difference between the voltage values was the result of a stronger illumination change in the projector setup. In contrast to the projector setup, where a projector was switched on, in the monitor setup only a black square on the normally illuminated monitor was removed. After the *z*-standardization, the normalized voltage values were once more baseline corrected, based on the same pre-display change interval of 100 ms prior to the crossing of the invisible boundary.

**Figure 5:**
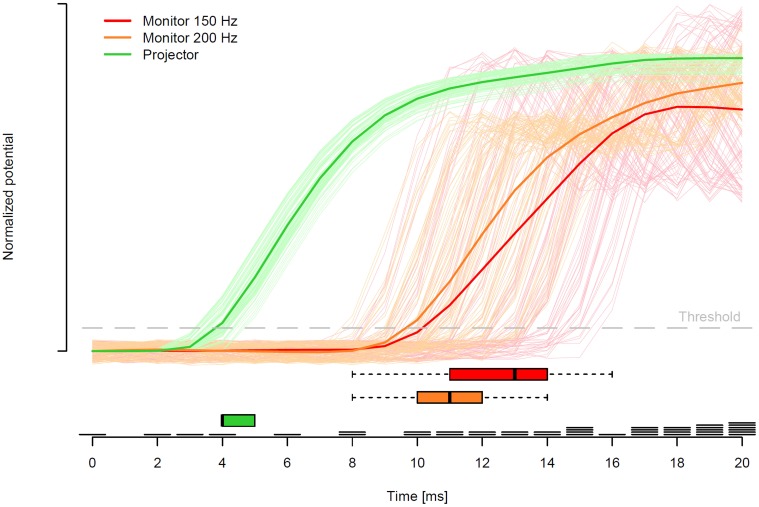
**Solid lines represent the mean of *z*-standardized voltage changes after baseline correction relative to the point in time when the invisible boundary was crossed**. Lines in light green, orange, and red indicate each single trial of the projector setup, the monitor setup with 200 Hz, and the monitor setup with 150 Hz, respectively. An increase in standardized voltage indicates a display change measured by the photosensitive diode. Red, orange, and green boxes-and-whiskers in the lower part indicate the median and 95% CI of the point in time when the voltage increased above the threshold (gray dashed line; 0.2 standardized voltage) for the two monitor and projector measurements, respectively. Finally, right above the time axis, exemplary eye movement data of one participant is presented, where each of the black bars represents the start of one fixation at this moment.

Figure 5 shows all single trial voltage changes from the two measurements of the monitor setup (light red for 150 Hz and light orange for 200 Hz) and the projector setup (light green) with the corresponding mean voltage changes in red, orange, and green. The light green lines, which correspond to one display change each in the projector setup, indicate relatively short display change latencies by a fast increase of mean and single trial voltages. Furthermore, the green lines indicate a low variability of the display change latencies in the projector setup. In contrast, in the monitor setup, the display change latencies of both refresh rates were prolonged and the variance of the single trials was markedly increased. Surprisingly, the two refresh rates of the monitor setup did not differ substantially, with the exception that the 150 Hz refresh rate tended to have especially prolonged display change latencies (up to 16 ms).

In the present investigation, we defined display change latency by the point in time when a threshold at 0.2 of standardized voltage was reached. This threshold (gray line in Figure 5) was selected in a way that the noise before the display change was not able to meet the voltage threshold. The boxes-and-whiskers, at the bottom of Figure 5, display the median (black vertical bar) and the 95% confidence interval of the display change latencies. The medians indicate that the majority of the display changes in the projector setup started with about 4 ms and in the monitor setup with 200 and 150 Hz the majority of the display changes started at 11 and 13 ms, respectively. In addition, a much larger deviation of display change latencies in both monitor setups in contrast to the projector setup was found. Note, for the projector setup we not only measured the latency for switching on the second projector but also the latency for switching off the first projector, which was highly comparable with a median latency of 4 ms.

Furthermore, each of the black horizontal bars right above the time axis of Figure 5 indicates the start of one fixation of the eye tracking measurement in the 200 Hz monitor condition. This exemplary eye movement data illustrates the importance of the fast display changes. In case that the projector setup was used to present the sentences, the number of trials in which the display change was too slow would be only about 4 out of 100 (4%). In contrast, the display changes were too slow in about 17 (17%) and 22 (22%) trials in the monitor setup with 200 and 150 Hz, respectively.

## Discussion

In the present paper, we compared a novel LED-based video projection system to a state-of-the-art CRT monitor in a gaze-contingent invisible boundary paradigm during sentence reading. As expected, the projector setup outperformed the monitor setup with respect to both median and range of display change latencies. Specifically, median latencies for the projector setup were about one-third of the median latencies for the monitor setup (4 ms compared to 11/13 ms). Furthermore, while the monitor setup latencies ranged up to 16 ms, the projector setup latencies did not exceed 5 ms. Thus, the very short display change latencies of the novel projector setup are much more likely to be finalized during a saccade compared to the display changes in the monitor setup. In the following, we will discuss some methodological considerations in gaze-contingent display change paradigms, the implications of our findings, and possible applications as well as practical aspects of the novel visual stimulation system.

Already two decades ago, it was argued that early implementations of gaze-contingent display change paradigms were likely to have suffered from technical problems, leading to the conclusion that results were partly not more than artifacts of the paradigm. Specifically, O'Regan (1990) proposed two kinds of technical problems related to stimulus presentation. First, because of temporal limitations of the eye tracking device, the experiment computer, and the refresh rate of the stimulus screen, display changes, which were intended to take place during a saccade, actually took place after the saccade ended. In other words, the display change actually took place during the time of the next fixation. The delayed display change resulted in a flicker or contrast change during the subsequent fixation, which may have influenced sensory information extraction and, in turn, affected eye movement behavior (e.g., fixation duration).

Second, older monitors suffered from prolonged persistence of CRT phosphors, leading to afterglow effects. These effects resulted in smearing and reduced contrast of subsequently presented stimuli. The proposed concerns were directly addressed by Inhoff et al. (1998), who measured eye movement behavior in gaze-contingent display change paradigms for four different screen refresh rates. Furthermore, they compared a phosphor-based CRT with an electroluminescent panel, which should not suffer from erosion of contrast. Inhoff et al. (1998) found no evidence that technical artifacts compromise the results of gaze-contingent display change paradigms unless atypically slow refresh rates or relatively slow phosphors are used. Therefore, with high eye tracker sampling rates (1000 Hz and above), fast computers, and fast CRT monitors, technical limitations regarding stimulus presentation should not be a problem nowadays.

Nevertheless, sometimes it happens that the timing is too slow and participants are able to detect gaze-contingent display changes. In an invisible boundary paradigm (involving a gaze-contingent display change), White et al. (2005) directly compared parafoveal preview effects in participants who were not aware of the change to effects in participants who were aware of the change. By using short and distinctive orthographically illegal previews (consonant strings), they increased the proportion of aware participants to one out of three. The results clearly demonstrated a qualitatively different pattern of eye movement data in readers who were aware of the display change compared to readers who were not aware. Thus, whether or not participants detect supposedly “invisible” gaze-contingent display changes has important implications for interpretation of the findings of such experiments.

As already mentioned in the Introduction, Slattery et al. (2011) showed that detection of gaze-contingent display changes does not only depend on the timing of the display change relative to the end of the saccade but also on the position of the pre-boundary fixation relative to the to-be-changed target and the nature of the experimental manipulation of the target. Specifically, Slattery et al. (2011) used a gaze-contingent invisible boundary paradigm and manipulated the delay of the display change (0, 15, 25 ms) as well as the properties of the parafoveal preview (letter identity or letter case change). They found a complex pattern evidenced by a marked interaction between the timing of the display change and the relationship between the preview and target characteristics. Importantly, even without an artificial delay in the display change, detection sensitivity was influenced by the amount and quality of information that was changed between the pre- and post-boundary stimulus. In addition, proximity of the pre-boundary fixation to the boundary influenced display change detection sensitivity. To put it simply, participants' sensitivity to detect the display changes depended on the when, where, and what of the display changes. This complex interaction can lead to significant artifacts in the eye movement data, which would distort the experimental effects, thereby rendering interpretation of findings extremely difficult.

Nearly all studies from the last years employing invisible boundary paradigms during reading used conventional CRT monitors with a refresh rate between 150 and 200 Hz. Moreover, they reported mean display change latencies between 5 and 10 ms with a range up to 20 ms but the procedure for measuring these display change latencies was hardly ever described. In many cases, it seems like display change latencies were solely estimated based on the refresh rate of the monitor (e.g., assumed 5 ms latency for a 200 Hz monitor). However, as shown by our diode-based measurements, such assumptions are invalid because they only consider the best-case scenario (i.e., the command to change the display is sent immediately before a screen refresh cycle) and they do not take into account the processing time of hardware (e.g., eye tracker) and software (e.g., Experiment Builder) components of the experimental setup. In our hardware-based measurements, we found a median latency of 11 ms for a 200 Hz monitor. Therefore, it is most likely that the display change latencies were actually longer than reported in the studies, which, in turn, may have affected the results of these studies. Note that the presently identified relatively long latencies in the CRT setup cannot be due to an artifact of our measurement circuit because the combination of a real-time photosensitive diode (with a rise time <1 ms) together with 1000 Hz EEG recordings (enabling a 1 ms resolution) led to reasonably short latencies in the projector setup. In the projector setup, in contrast to the monitor setup, we found short latencies with little variability (range 4–5 ms).

As evidenced by the results of our measurements, the LED-based projector system provides a reliable tool for fast display changes. In so doing, it replaces slower and less reliable conventional CRT monitors, which are based on outdated technology. Today, many labs face the practical problem that they run out of reasonable visual stimulation equipment because CRT monitors have almost vanished from the market and widely available LCD monitors are limited to mostly 60 Hz, which are likely to result in much slower gaze-contingent display changes. Even modern projector systems with a refresh rate up to 120 Hz and recently developed gaming monitors with up to 144 Hz might be too slow for precise gaze-contingent display changes as they still require the construction of a new display, which involves several time-consuming processes (e.g., the response time of the monitor). In contrast, by switching between two already constructed displays, our projector system bypasses the construction of a new display. By offering a future-proof, advanced alternative based on LED technology, our system provides a more than feasible solution for precise visual stimulation.

Besides application in conventional eye tracking experiments, our system is particularly suitable for experiments with simultaneous registration of eye movements and brain electrophysiology. Brain electrophysiology (as measured by EEG or MEG) is extremely susceptible to visual stimulation. Therefore, even if a display change that takes place during a fixation is not consciously detected by the participant, it is highly likely that such a display change interferes with visual information extraction and, as a consequence, affects brain activity measures (e.g., event-related potentials). Therefore, rigorous control over exact timing of visual stimulation is absolutely necessary in experiments that combine eye tracking and brain activity methods. Besides application in electrophysiological studies, our system is especially suitable for visual presentation in fMRI studies. Functional MRI was previously excluded from high-speed visual stimulation because it is not possible to operate conventional CRT monitors in the strong magnetic field of an MRI scanner. In addition, as already mentioned, present LCD technology as implemented in MR-compatible monitors and video projectors is limited to a rather slow refresh rate of 60 Hz, which excludes fast and exactly timed visual presentation. Therefore, the present LED-based video projection system offers new possibilities for combined eye tracking and fMRI studies using gaze-contingent display change paradigms and other experiments that require fast and reliable visual presentation.

Possible applications of our projector system not only include invisible boundary experiments but also subtle temporal manipulations in short-time presentation or masked priming studies. Specifically, it should be possible to implement fine-grained variations of visual presentation durations with previously unrivaled precision. Certainly, these benefits are not limited to experiments in the domain of reading research but may also be relevant for other fields like visual object processing, attention, search, and scene perception.

Despite the potential benefits of our system, there are a number of issues that have to be carefully addressed by experimenters. The positioning of the two projectors requires delicate alignment of the respective projection areas. Once perfect pixel-to-pixel alignment is achieved, the projectors should be protected from further movement (e.g., by mounting in a solid box or cage). In addition, there should be a standard routine for checking the alignment of the projectors before data from participants is acquired. A further issue is related to matching of color and brightness of the two projectors. Although the two projectors are identical models from a single manufacturer, there are slight differences in these parameters. The differences have to be accommodated for by manual adjustment via the projectors' built-in settings. In our case, we used black on white presentation, thereby keeping the need to accommodate for color to a minimum. Moreover, brightness of each projector was set to 490 lx (as measured by a light meter). On the positive side, there is no measurable dimming or brightening on the screen when the projectors switch. Furthermore, there are no special demands on the computer's graphics card (a single VGA output is sufficient) and there is no extra software required (the TTL triggers can be sent by the Experiment Builder software). In principle, our system could be used for more than one display change per trial, but the temporal limit for every second change is determined by the projectors' refresh rate (60 Hz in our case).

## Conclusion

The present video projection system provides a solution for high-speed visual stimulation as required by many psychological and neuroscientific experiments. Because it is based on projectors, it may be used not only for behavioral, eye tracking, and electrophysiological studies but also for fMRI studies. By enabling high-temporal precision of display changes, it facilitates the realization of gaze-contingent paradigms and other time-sensitive visual experiments. In addition, our system offers completely novel possibilities for such experiments in fMRI.

### Conflict of interest statement

The authors declare that the research was conducted in the absence of any commercial or financial relationships that could be construed as a potential conflict of interest.
